# Inadvertent Thyroid Radiation During Computed Tomography of the Chest: A Retrospective Study

**DOI:** 10.4314/ejhs.v33i2.17

**Published:** 2023-03

**Authors:** Hermon Miliard Derbew, Tesfaye Kebede, Seife Teferi, Hansel J Otero

**Affiliations:** 1 Department of Radiology, College of Health Sciences, Addis Ababa University, Addis Ababa, Ethiopia; 2 Children's Hospital of Philadelphia, Department of Radiology, Philadelphia, PA, USA

**Keywords:** Thyroid, radiation, Computer Tomography, CTDIvol, DLP, Effective Dose

## Abstract

**Background:**

The thyroid, along with the breast, lung, and bone marrow, is among the most radiosensitive organs. This study aims to assess the rate of unnecessary radiation exposure to the thyroid gland in patients who had chest Computed Tomography (CT) at a large teaching hospital.

**Method:**

Hospital-based retrospective cross-sectional study on 1,306 patients who underwent chest CT from July 2018 to January 2019. Thyroid gland inclusion along with the CT dose of the studies was evaluated. Data was collected by evaluating chest CT scans from Picture Archive and Communication System (MedWeb).

**Result:**

Out of 1306 patients, who had Chest CT scans intravenous iodinated contrast media was used in 95.4% of the CT scans. The thyroid was included in 99.8% of the scans, out of which 76.9% included the whole thyroid gland. Among the patients who had previous CT scans, 75.3% had one previous scan and 24.7% had two previous scans. DLP (Dose Length Product) in mGycm was lower in females (360.33±32) compared to males (426.45±378.4). The lowest DLP value was observed in the pediatric patients in the age range of 1–5 years which was 146.83, while the highest was observed among those above 18 years of age with mean DLP of 418.31.

**Conclusion:**

The majority of chest CT scans unnecessarily include the whole thyroid gland, which is one of the most sensitive organs for radiation-induced effects. Authors recommend optimized technique for chest scans to avoid future impacts.

## Introduction

The thyroid, breasts, lungs, and bone marrow are more sensitive to radiation because the cells in those areas divide rapidly (1). The association between radiation exposure and the occurrence of thyroid cancer has been well documented, and the two main risk factors for the development of a thyroid cancer are the radiation dose delivered to the thyroid gland and the age at exposure. The risk is more significant during childhood and decreases with increased age at exposure, being low in adults. After exposure, the minimum latency period before the appearance of thyroid cancers is 5 to 10 years (2). Mathews et al (3) compared cancer incidence in children aged 0 to 19 years exposed to CT scans between 1985 and 2005 with incidence in the corresponding unexposed population. The overall incidence of cancer was found to be 24% higher in the exposed group; risk increased with dose and with younger age at exposure.

Research by Baker and Bhatti in 2006 (4) stated that the rapid increase in CT use since 1990 and especially in the past 10 years has been accompanied by a coterminous worldwide increase in incidence of thyroid cancer, especially in women. While modern scanners are able to perform thoracic CT for wide range of clinical indication with increasingly low radiation dose (5), a multi-institutional study in the late 1990s, found effective doses for chest CT between 6–18 mSv (6). In a similar study in 2008, Chest CT effective dose ranged from 4–18mSv (7). Effective dose, a dosimetry quantity useful for comparing the overall health effects of nonuniform exposure in terms of an equivalent to whole body exposure, has a unit of Sievert (8).

The use of iodinated contrast with CT was also shown to increase the radiation absorbed by the thyroid by up to 35% (9). Chest CT scans caused higher thyroid dose and the LAR (Lifetime Attributable Risk) of thyroid cancer incidence, compared with paranasal sinus or head CT scans. Therefore, attention should be given to protect the thyroid during CT scans especially Chest CT (10). The aim of this study is to assess the rate of unnecessary radiation exposure to the thyroid gland and to assess the effective dose value of Chest CT scans at Tikur Anbessa Specialized Hospital (TASH) which is the largest tertiary and teaching hospital in Addis Ababa, Ethiopia.

## Methods and Materials

This is a hospital based retrospective cross-sectional study conducted on 1,306 patients who underwent a Chest CT scan between July 2018 and January 2019 at Tikur Anbessa Specialized Hospital (TASH). The study was approved by the Research and Ethics Committee. The sample included all patients who had chest CT during the study period. We excluded patients who had multiple region scan (eg. Chest with abdominopelvic CT scan), previous thyroid surgery, underwent the scan to evaluate the thyroid gland, have missing dose variables (DLP, Computed Tomography Dose Index Volume (CTDIvol and/or mean CTDI) and/or those who underwent CT for CT guided chest biopsy.

All studies were performed in a 64-row detector scanner (GE optimal, 8ikwas GE) and was retrieved from the Picture Archive Communication System (MedWeb) by a radiologist (H.M.D). The recording tool was designed by a radiologist. In order to evaluate the clarity of the findings, unclear images were evaluated with senior colleague with 15 years' experience (T.K). The data retrieval form included demographic, use of contrast, previous imaging, the inclusion of the thyroid in the images, and CT dose information (CTDIvol, scanning length, DLP). The effective dose (ED in mSv) was then derived using the product of DLP and (gender/age specific) published conversion factor (k) and DLP is the product of CTDIvol (mGy) and scanning length (l in cm) (11, 12).

**ED = DLP x k = CTDIvol x l x k:**

The k - factor value was published as ICRP 60 and 103 with the latter being published later, therefore we will be using that conversion factor in our study (11). The data was then checked for clarity and completeness. Data was analyzed using nonparametric statistical methods **and one-way ANOVA** with SPSS version 24.0 software package.

**Ethics Approval:** In order to respect the patient's bill of rights, ethical considerations were taken into account. This is a retrospective study where patient identifiers were not used in the data collection or analysis. Any piece of information was kept confidential by keeping anonymity of the study subjects.

## Results

**Figure 1:**
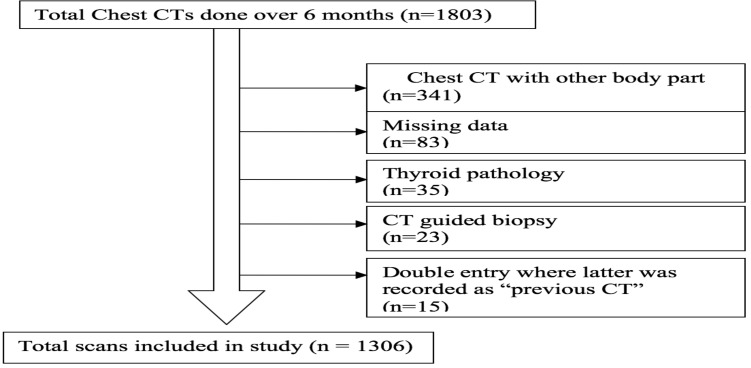
The number of total CT scans included in the study after exclusion criteria applied

A total of 1803 CT studies were identified, with 1306 in the final sample after exclusions (Figure 1).

The mean age was 41.7 years (SD ±18.6). Age categorization showed most (89.9%) were adults (age >18 years). Male and female proportion was comparable with male accounting for 666 (48.9%) of the total (Table 1). Malignancy related causes for scanning accounted for 59.6% of reasons Chest CT was requested.

**Table 1:** Demographic characteristics

Demographics (n=1306)	
**Age (years)**	**41.7 ± 18.6**
**Sex (girls:boys)**	**(1:1.04)**
**Indication**	
Malignancy related	778
Cough	225
Fever	79
Chest pain	67
Hemoptysis	44

**Figure 2:**
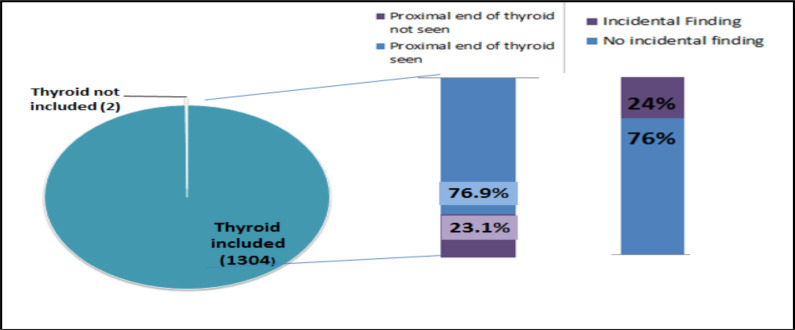
Rate of Thyroid Inclusion during CT scan and Incidental Finding on thyroid.

Intravenous iodinated contrast media was used in 95.4% of the CT scans, with 60.7% being post contrast only and the rest 34.7% had both pre and post contrast CT scan. Except in two patients, thyroid was included in all the remaining patients (99.8%). The whole thyroid gland was involved in 76.9% of patients, as evidenced by visualization of the upper lobe of thyroid gland (fig 2). Incidental thyroid pathology was seen in 312 patients (24%).

As shown in Table 2, 12.6% of the patients had one or more previous CT scans, with 75.3% having one previous CT scan and 24.7% having two or more previous CT scans. Similar to the current CT scan, 95% of the previous CT were contrast enhanced, thyroid was involved in 98.7% of the previous scans, with 69.9% involving the whole thyroid gland.

**Table 2:** Type of CT scan, involvement of thyroid gland and previous CT assessment

Parameter	N (%)
**Type of CT**	
Precontrast	60 (4.6%)
Post contrast	793 (60.7%)
Both	453 (34.7%)
**Thyroid Included**	
Yes	1304 (99.8%)
No	2 (0.2%)
**Proximal End of Thyroid Seen**	
Yes	1001 (76.9%)
No	301 (23.1%)
**Incidental Finding**	
Yes	312 (24.0%)
No	989 (76.0%)
**Previous CT**	
Yes	164 (12.6%)
No	1140 (87.4%)
**Type of Previous Ct**	
Precontrast	8 (5.1%)
Post contrast	104 (66.7%)
Both	44 (28.2%)
**Thyroid Included in Previous CT**	
Yes	151 (98.7%)
No	2 (1.3%)
**Proximal End of Thyroid Seen**	
Yes	107 (69.9%)
No	46 (30.1%)
**How Many Previous CT**	
1	119 (75.3%)
≥2	39 (24.7%)

**Radiation dose assessment**: The correlation of scan length with age and sex was assessed. The person's correlation coefficient for age was 0.33 and for sex was -0.12. This indicates that the correlation of scan length with age and sex was weak; as age increases scan length will increase and female have lower scan length than male. The largest mean scan length of 411mm (SD±43.25) was seen in patients with age greater than 18 yrs. Age was also classified in different categories and the respective mean scan length in each age category was done by using ONE WAY ANOVA (analysis of variance).

DLP was lower in females (360.33±329.4) mGy-cm compared to males (426.45±378.4)mGy-cm.; this difference was statistically significant with a mean difference of 134. The lowest DLP value was observed in the pediatric patients in the age range of 1–5 years which was 146.83 (SD±103.9) mGy-cm, while the highest was observed among those >18yrs with the mean being 418.31mGycm(SD±364.8). The estimated mean effective dose of chest CT scan for patients above 18 years using ICRP 103 conversion factor was 8.7845mSv (SD ±7.66169) (Table 3).

**Table 3:** Mean and third quartile distributions of Effective Dose, Scan Length, CTDIvol and DLP in age and sex categories

	Mean CTDIvol (mGy)		DLP (mGy.cm)		ED (mSv) ICRP103(11)
	Mean ± SD	3^rd^ Quartile (Q3)	Mean ± SD	3^rd^ Quartile	Mean ± SD
**Sex**					
Male	7.9 ± 4.7	9.9	426.5 ± 378.4	538.0	9.5 ± 8.9
Female	7.1 ± 6.7	8.5	360.3 ± 329.4	455.7	7.8 ± 7.0
**Age**					
<1yr	5.5 ± 1.4	6.7	295.0 ± 414.2	403.3	29.2 ± 41.0
1–5yr	5.5 ± 1.7	7.1	146.8 ± 103.9	169.9	9.4 ± 6.6
5–10yr	6.5 ± 3.2	7.8	228.2 ± 163.5	294.8	10.7 ± 7.7
10–15yr	4.2 ± 2.4	6.9	154.1 ± 124.9	203.5	5.1 ± 4.1
15–18yrs	4.6 ± 2.3	5.2	190.5 ± 131.7	215.8	4.6 ± 3.2
≥18yrs	7.8 ± 6.0	9.8	418.3 ± 364.8	528.5	8.8 ± 7.7

Multivariate Linear logistic regression of variables with DLP							

	Unstandardized Coefficients		Standardized Coefficients	t	Sig.	95.0% Confidence Interval for B	
				
	B	Std. Error	Beta			Lower Bound	Upper Bound
(Constant)	-148.048	60.194		-2.460	.014	-266.135	-29.961
Scan Length	.475	.137	.079	3.468	.001	.206	.743
Mean CTDIvol	32.780	1.338	.537	24.491	.000	30.154	35.406
Age	4.084	.446	.213	9.155	.000	3.209	4.959
Sex	-42.948	15.345	-.060	-2.799	.005	-73.052	-12.844

Among the variables, CTDIvol was the parameter that was strongly correlated with DLP value, with Pearson correlation coefficient of 0.54. An increase in CTDIvol by one unit will increase the DLP by almost 32 times (95% CI 30.15–35.41). The variables scan length, age, and sex; even though were associated with DLP value, they were weakly correlated with DLP value and had poor estimation power as evidenced by very low correlation coefficient, larger SE and wider confidence intervals (Table 3).

The value of ED was significantly and independently associated with mean CTDIvol, scan length, and age of the patient, whilst sex of the patient was not associated with the amount of ED. The amount of the mean CTDIvol was the main determinant factor for the observed variation in the amount of ED (Table 4). Among the age groups, the highest ED was seen in children under 1 year of age while the lowest was in adolescents in the age range 15–18years. The estimated mean effective dose of chest CT was 8.66mSv (SD ±8) and the proportion of patients exposed to ED higher than 9mSv and 20mSv were 34.3% and 7%, respectively (Table 4).

**Table 4:** Multivariate Linear logistic regression of determinant factors for Effective Dose

Model	Unstandardized Coefficients		Standardized Coefficients	t	Sig.	95.0% Confidence Interval for B	
					
	B	Std. Error	Beta			Lower Bound	Upper Bound
Mean_CTDIvol	.786	.032	.630	24.610	.000	.723	.848
Scan_Length	.027	.004	.936	7.438	.000	.020	.035
Sex	.149	.353	.020	.422	.673	-.544	.842
Age	-1.476	.282	-.725	-5.228	.000	-2.029	-.922

## Discussion

The optimal scan for a routine chest CT is to scan from thoracic inlet cranially to include upper abdomen caudally. The thyroid gland, because of its location spanning from C5 to T1 with the apex of the lung being at T1 vertebral level (13), may compel the lower lobe of the thyroid gland for inadvertent radiation exposure during chest CT scan due to overlap. However, in our study, thyroid gland was included in 99.8% of the scans with 76.9% having the whole of the thyroid included. 12.6% of the patients who were scanned previously in addition to the current one, had thyroid included in 98.7% with 69.9% having proximal end visualized. In addition, 95.6% of the scans utilized iodinated contrast media which further increases the risk of radiation associated toxicities. A study showed that the use of iodinated contrast with CT increases the radiation absorbed by the thyroid by up to 35% (9).

Regarding radiation dose assessment, the mean ED (using ICRP 103) was 8.66mSv with 7% having ED greater than 20mSv which is higher than effective dose found in a study done in Netherlands which showed a range of 6–18 mSv(6). In addition, the risk of developing thyroid cancer from radiation was found to be more significant during childhood whilst decreasing with increased age at exposure, being low in adults (2). In our study, we found that among the age groups, the highest ED was documented in children under 1 year of age.

Therefore, what can be done to decrease radiation induced damage to the thyroid? A study done in 2011 at Duke University using MOSFET (metal oxide semiconductor field effect transistor) showed that thyroid radiation dose decreased by 28%, 33%, and 45% by organ-based dose modulation, automatic tube current modulation with thyroid shield, and organ-based dose modulation with thyroid shield protocols, respectively, compared with the use of automatic tube current modulation alone (p ≤ 0.005) (14). In addition, since the mean CTDIvol was the most determinant factor for variation in the observed ED, adjusting the parameters that in turn determine the amount of the CTDIvol, such as the KV will regulate the radiation received, especially in children (<1year) where the highest mean ED was recorded. Moreover, the scan length, which was found to be significantly and independently associated with ED, should also be selected appropriately based on the diagnostic information that is provided.

The appearance of CT imaging does not help to estimate the possibility of excess radiation exposure. In fact, the quality of CT imaging will improve as the dose of radiation is increased. There will always be a tradeoff between the quality of the image and the risk of radiation. It is the responsibility of the radiologist and radiographer to decide the imaging protocol that should be used in a particular disease and patient conditions with the aim of achieving the required diagnostic information by using the minimum possible radiation dose (ALARA principle).

Some limitations in our study included incomplete dose information in some studies, documentation for indication had specific diagnosis rather than clinical symptoms, images taken prior to April 2017 (date MedWeb was installed) and images in other facilities (other than TASH) were unavailable. In conclusion, anatomically, the apex of the lung coincides with the lower end of thyroid making part of thyroid inclusion unavoidable. However, 76.9% of the scans included the whole of thyroid gland, which technically could be avoided. Although the mean ED (calculated with ICRP 103) was within the range of the recommended limits for chest CT scan, however, 7% of the cases were exposed to higher than the recommended (>20mSv) ED. In addition, the highest ED was seen in children under 1 year of age which proves how radiation conscious one has to be whilst radiating a child since the risk of developing thyroid cancer due to radiation is highest when exposure is in childhood as compared to adulthood (2). Furthermore, the use of organ-based dose modulation with thyroid shield protocols has shown to decrease radiation to thyroid by 45% (14).
